# Theta-Band Oscillations as an Indicator of Mild Traumatic Brain Injury

**DOI:** 10.1007/s10548-018-0667-2

**Published:** 2018-08-10

**Authors:** Hanna Kaltiainen, Liisa Helle, Mia Liljeström, Hanna Renvall, Nina Forss

**Affiliations:** 1 Department of Neuroscience and Biomedical Engineering, School of Science, Aalto University, P.O. Box 12200, 00076 Aalto, Espoo, Finland; 2 Aalto Neuroimaging, MEG Core, Aalto University, Espoo, Finland; 3 Department of Neurology, Lohja District Hospital, Sairaalatie 8, 08200 Lohja, Finland; 4 Elekta Oy, P.O. Box 34, 00531 Helsinki, Finland; 5 Clinical Neurosciences and Department of Neurology, University of Helsinki and Helsinki University Central Hospital, P.O. Box 340, 00029 HUS, Helsinki, Finland

**Keywords:** Low frequency activity, Traumatic brain injury (TBI), Magnetoencephalography (MEG), Repeated measurements, Resting-state, Oscillations

## Abstract

Mild traumatic brain injury (mTBI) patients continue to pose a diagnostic challenge due to their diverse symptoms without trauma-specific changes in structural imaging. We addressed here the possible early changes in spontaneous oscillatory brain activity after mTBI, and their feasibility as an indicator of injury in clinical evaluation. We recorded resting-state magnetoencephalography (MEG) data in both eyes-open and eyes-closed conditions from 26 patients (11 females and 15 males, aged 20–59) with mTBI 6 days–6 months after the injury, and compared their spontaneous oscillatory activity to corresponding data from 139 healthy controls. Twelve of the patients underwent a follow-up measurement at 6 months. Ten of all patients were without structural lesions in MRI. At single-subject level, aberrant 4–7 Hz (theta) band activity exceeding the + 2 SD limit of the healthy subjects was visible in 7 out of 26 patients; three out of the seven patients with abnormal theta activity were without any detectable lesions in MRI. Of the patients that participated in the follow-up measurements, five showed abnormal theta activity in the first recording, but only two in the second measurement. Our results suggest that aberrant theta-band oscillatory activity can provide an early objective sign of brain dysfunction after mTBI. In 3/7 patients, the slow-wave activity was transient and visible only in the first recording, urging prompt timing for the measurements in clinical settings.

## Introduction

The diagnosis of mild traumatic brain injury (mTBI) remains a challenge, and despite extensive remaining symptoms many of these patients fail to show trauma-specific changes in structural imaging such as magnetic resonance imaging (MRI) or computed tomography (CT). Currently, MRI is performed only in a minority of cases particularly within the acute phase when trauma-specific changes are most readily identified (Brandstack et al. 2006). Even when visible, trauma-specific changes correlate poorly with symptoms (Jacobs et al. 2010; Lee et al. 2008). Prolonged symptoms lasting over 3 months post-injury are present in approximately 5–20% of mTBI patients, while some remain symptomatic for years (Iverson et al. 2012; Losoi et al. 2016). So far, there are no unequivocal biomarkers of mTBI available, and diagnostic criteria are currently based on clinical patient history (The American Congress of Rehabilitation Medicine Committee on Social, Ethical, and Environmental Aspects of Rehabilitation 1993; Carroll et al. 2004; Levin and Diaz-Arrastia 2015).

Low-frequency activity (~ 0.5–7 Hz) measured with electroencephalography (EEG) or magnetoencephalography (MEG) is a frequent finding in patients with varying brain pathologies as well as in TBIs (Oppenheimer 1968; Gloor et al. 1977; Lewine et al. 1999, 2007; Huang et al. 2009, 2014), whereas they are rare in a healthy adult brain (Kaltiainen et al. 2016). Lewine et al. (2007) demonstrated MEG low-frequency activity in 14/20 mTBI patients measured 2–38 months after trauma, and Huang et al. (2009) found abnormal activity at 1–4 Hz in 10/10 symptomatic mTBI patients 1–46 months after the insult. Huang et al. (2009) compared the low-frequency activity in MEG measurements with underlying deficits in diffusion tensor imaging tractography, and found MEG more sensitive in detecting lesions. Behaviorally, low-frequency activity measured with MEG at 3 months after trauma has, at group level, been associated with problems in neuropsychological functioning in mTBI (Swan et al. 2015), and reduced connectivity at low-frequency band correlated with neuropsychological improvement after rehabilitation in severe TBI patients (Castellanos et al. 2010).

It has, however, remained unclear whether low frequency activity measured at subacute stage after mTBI could qualify as a useful early measure in predicting those patients who will get prolonged symptoms. Geets and Louette (1985) demonstrated slowing of EEG in 2–17% of young adult TBI patients, depending on the severity of the concussion, measured within 48 h after the trauma. Bierbrauer et al. (1992) measured TBI patients at multiple time points, starting from the acute phase, without any control group. Aberrant EEG activity was observed in 82% of the patients at the initial evaluation, in 50% the EEG returned to normal limits within 8 weeks. On the basis of the duration of unconsciousness, part of the patients in both of these studies would nowadays be considered moderate TBIs. However, the predictability of long-term symptoms appears particularly problematic in patients suffering from mild TBIs that were targeted in the present study.

We have earlier collected resting-state MEG data from 139 healthy control subjects without any neurological or neuropsychiatric problems or medications (Kaltiainen et al. 2016). In that group, only two subjects (1.4%) showed aberrant low-frequency oscillatory activity. Here we present MEG recordings of oscillatory activity from 26 mTBI patients and compare them with the control group. Importantly, 13 patients were measured within 4 weeks of the trauma. For evaluating the method’s sensitivity for clinical diagnostic purposes, we included only patients with first-ever mTBI, in contrary to earlier studies addressing also patients with a history of multiple mTBIs (Huang et al. 2009). To observe the evolvement of low-frequency activity during recovery, 12 patients with mTBI underwent follow-up measurements at 6 months after the injury.

## Materials and Methods

### Subjects

We analyzed spontaneous oscillatory activity from 26 patients (11 females, 15 males) with traumatic brain injury. Thirteen subjects were measured within 4 weeks of the head trauma (see Table 1), and 12 patients underwent a follow-up measurement at 6–7 months. Patients were 20–59 years old (average ± standard error mean (SEM) 41 ± 2 years.; females 44 ± 3 years., males 41 ± 3 years., Table 1). All patients gave their informed consent to participate in this study. The study was accepted by Ethic Committee of Helsinki and Uusimaa Hospital District. Demographics of the patients are described in Table 1. The control group consisted of 139 healthy subjects (102 females and 37 males, aged 18–60, average ± SEM 31 ± 1 years., Table 1), described in detail in our previous study (Kaltiainen et al. 2016). All controls had received a questionnaire inquiring history of mild head traumas and hobbies with high susceptibility to mTBI, and those with positive mTBI history were excluded.

**Table 1:** Age and gender distribution of the patients and controls

Age	18–29	30–45	46–60	all (18–60)
Patients				
Male	3/20%	7/47%	5/33%	15/58%
Female	2/18%	4/36%	5/46%	11/42%
All	5/19%	11/42%	10/39%	26
Controls				
Male	6/16%	15/41%	6/16%	37/27%
Female	59/58%	32/31%	11/11%	102/73%
All	75/54%	47/34%	17/12%	139

### Clinical Evaluation

All patients had their first ever TBI and were without any history of neuropsychological disorders, medications or substance abuse. They fulfilled American Congress of Rehabilitation Medicine (ACRM) criteria for mTBI with trauma-induced alteration of mental state at the time of the accident and loss of consciousness for < 30 min (The American Congress of Rehabilitation Medicine Committee on Social, Ethical, and Environmental Aspects of Rehabilitation 1993). Glasgow coma scale (GCS) addresses the level of consciousness and varies between 3 (deep unconsciousness) and 15 (alert and oriented) (Teasdale and Jennett 1974). GCS of the patients varied between 13 and 15 at 30 min after trauma or later (see Table 2), thus fulfilling the GCS criteria of mTBI (The American Congress of Rehabilitation Medicine Committee on Social, Ethical, and Environmental Aspects of Rehabilitation 1993; Carroll et al. 2004). All patients maintained TBI symptoms at the time of the first MEG measurement session, and to assess the remaining symptoms at each measurement session they filled in the Rivermead Post-Concussion Symptom Questionnaire (RPCSQ) questionnaire (Eyres et al. 2005; King et al. 1995). The RPCSQ is commonly used to describe subjective symptoms after TBI: it addresses the occurrence of 16 cognitive, emotional and somatic post-concussive symptoms, and their severity with a five-step scale. Wilcoxon two-related-samples test assessed the possible changes in RPCSQ score between two consecutive MEG-measurement sessions.

**Table 2:** Demographics of the patients: age at the time of injury, initial GCS, timing of MEG-measurements and MRI (after injury), trauma lesions in MRI and low-frequency activity in MEG-measurements, results of Rivermead Post-Concussion Symptoms Questionnaires

Patient	Age	GCS	MEG1	MEG2	MRI	MRI lesion	MEG1: LFA	MEG2: LFA	RMPCQ1	RMPCQ2
1	43	15	4 months		16 months	−	+		3	
2	50	15	2 months		12 months	+/s	−		3	
3	42	14	5 months		1 week	+/d	−		24	
4	46	14	4.5 months		4 months	+/d	−		29	
5	37	14	3.5 months		8 months	+/d	+		13	
6	32	15	4 months		1 week	+/d	−		18	
7	50	15	2 months		9.5 months	+/d	−		24	
8	59	15	3 weeks		3 weeks	+/s	−		3	
9	54	15	2 months		1.5 months	−	−		8	
10	39	15	2 months		1 month	−	−		31	
11	20	14	1 month		1 week	+/d	−		2	
12	44	14	1.5 months		3 months	+/s	−		27	
13	43	14	6 months		6 months	−	+/slow alpha		28	
14	36	14	1.5 months		2 weeks	+/d	−		25	
15	39	15	3 weeks	7 months	3 weeks	−	+	−	9	7
16	29	14	1 month	6 months	1 week	−	+	+	3	2
17	37	14	1 month	6 months	1.5 months	+/d	+/slow alpha	+/slow alpha	25	14
18	50	14	2 months	6 months	1 month	+/s	+	+	6	3
19	28	15	1 week	6 months	3.5 weeks	−	−	−	16	14
20	29	14	3 weeks	6 months	1 month	+/d	-	−	3	2
21	59	14	1 week	6 months	1 month	+/s	+	−	36	18
22	53	14	3 weeks	6.5 months	1 week	+/d	−	−	34	6
23	51	15	1 week	6 months	2 weeks	−	−	−	14	6
24	23	15	1 week	6 months	1 month	−	−	−	25	0
25	40	14	1 month	7 months	2 months	+/s	+	−	14	3
26	56	15	3 weeks	6 months	2 months	−	−	−	32	16

*GCS* Glascow Coma Scale, *LFA* low-frequency activity, *RMPCQ* Rivermead Post-Concussion Symptoms Questionnaire, *d* deep lesion, *s*  superficial lesion

### MEG and MRI Data Collection

Measurements took place at Aalto Neuroimaging, MEG Core, Aalto University School of Science, Espoo, Finland, with a 306-channel whole-head MEG device (Elekta Neuromag™, Elekta Oy, Helsinki, Finland) in a magnetically shielded room. The device consists of 102 triple sensors with two orthogonal planar gradiometers and one magnetometer, each coupled to a Superconducting Quantum Interference Device sensor. Five head position indicator (HPI) coils attached to the scalp and measured with a 3D digitizer (Polhemus 3Space® Fastrak™, Colchester, VT, US), indicated the head position in relation to the sensor helmet during each measurement session. Digitization of the two preauricular points and the nasion anchored the head coordinate frame. Horizontal and vertical electro-oculogram (EOG) as well as electrocardiogram (ECG) were measured for artefact management. Recording of MEG signals occurred with a sampling rate of 1000 Hz and a band-pass filter of 0.03–330 Hz. For sensor-level analysis, down-sampling the data offline to 200 Hz enabled comparison with the previously measured control data, resulting in a frequency band of 0.03–67 Hz.

Recordings consisted of 10 min of eyes-open and eyes-closed condition each. During the measurements, the patients sat relaxed, avoided any movements and fixated at a fixation cross during the eyes-open recordings. A short pause at 5 min during the eyes-open recordings and two short pauses during the eyes-closed recordings were introduced to assure that the subjects were awake and alert.

All patients underwent the first MEG measurements within 6 months after the injury; 13 patients were measured within 4 weeks of the trauma (see Table 2). Twelve of the patients had a follow-up measurement at 6 months; their first measurement occurred within 6 days–2 months after the injury. The second measurement thus followed at 6–7 months after the injury.

1.5 T MRI anatomical scans (GE Signa HDX 1.5 T, Milwaukee, Wisconsin) were obtained from all patients at one week to 16 months after the injury. The imaging included sagittal T2 Cube, fluid attenuated inversion recovery, axial fast spin echo, axial #D susceptibility weighted imaging and axial 3D fast SPGR T1 sequences.

### Data Analysis

#### Preprocessing

Many of the lesions observed after TBI typically reside in regions sensitive to physiological artefacts in EEG and MEG measurements, such as the orbitofrontal cortex (eye-blink artefacts) and the basal temporal and occipital areas (cardiac artefacts). For separating brain activity from external and near-by artifacts we used the temporal extension of the signal space separation (SSS) method (Taulu and Simola 2006; MaxFilter™ software, Elekta Oy, Helsinki, Finland). Independent component analysis (Hyvarinen and Oja 2000) served to identify and project out 1–2 of the most prominent cardiac QRS-complex -related components, as well as the most prominent component related to eye blinks. To allow sensor-level comparison between individual patients and control subjects, an SSS-based head position transformation algorithm transferred the individual patient measurements to the average head position calculated over control subjects (Taulu et al. 2004).

#### Spectral Estimation

After preprocessing, we estimated the individual sensor-level amplitude spectra for data downsampled to 200 Hz using Welch’s method, with a 1024-point fast Fourier transform (FFT) with 50% overlap and Hann windowing. The low-frequency ranges (0.5–7 Hz) of each patient’s spectra were then compared with the average spectra calculated over the control subjects (Kaltiainen et al. 2016). We first analyzed the data from the eyes-closed condition and continued with the eyes-open data if activity exceeding the + 2 SD limit of the healthy control group was observed.

#### Source Modelling

In patients with aberrant low-frequency activity compared with the control subjects, source-level power spectral densities were estimated from the data using the MNE-Python package with Welch’s method (8196-point FFT with 50% overlap and Hann windowing, data sampling rate 1000 Hz) (Gramfort et al. 2014). Source localization was performed using cortically constrained L2 minimum-norm estimate (MNE) (Gramfort et al. 2013; Hamalainen and Ilmoniemi 1994). For this, FreeSurfer was used to segment the cortical mantle and cranial volume from anatomical T1 MR images (Fischl et al. 2002). Noise-normalized MNEs (dynamical Statistical Parametric Maps, dSPMs) were calculated over the cortex for estimating the signal-to-noise ratios in each potential source location (Dale et al. 2000). The noise covariance matrix was estimated from empty room measurements obtained in the same or a recent measurement session.

#### Effect of Source Depth

The main contributor to MEG’s sensitivity in detecting neural activity is the depth of the current source (Hillebrand and Barnes 2002). To estimate the effect of lesion depth on detecting low-frequency MEG activity, we related the MEG findings with the lesion depth (superficial or deep) in patients with detected structural lesions. Lesions with depth of < 3 cm were defined as superficial. Chi square statistic served for comparing the occurrence of low-frequency activity between patients and controls, and with deep versus superficial MRI lesions. Mann–Whitney U two-independent-samples test provided comparison of RPCSQ scores between patients with or without MRI and/or MEG findings.

## Results

### MEG

**Fig. 1:**
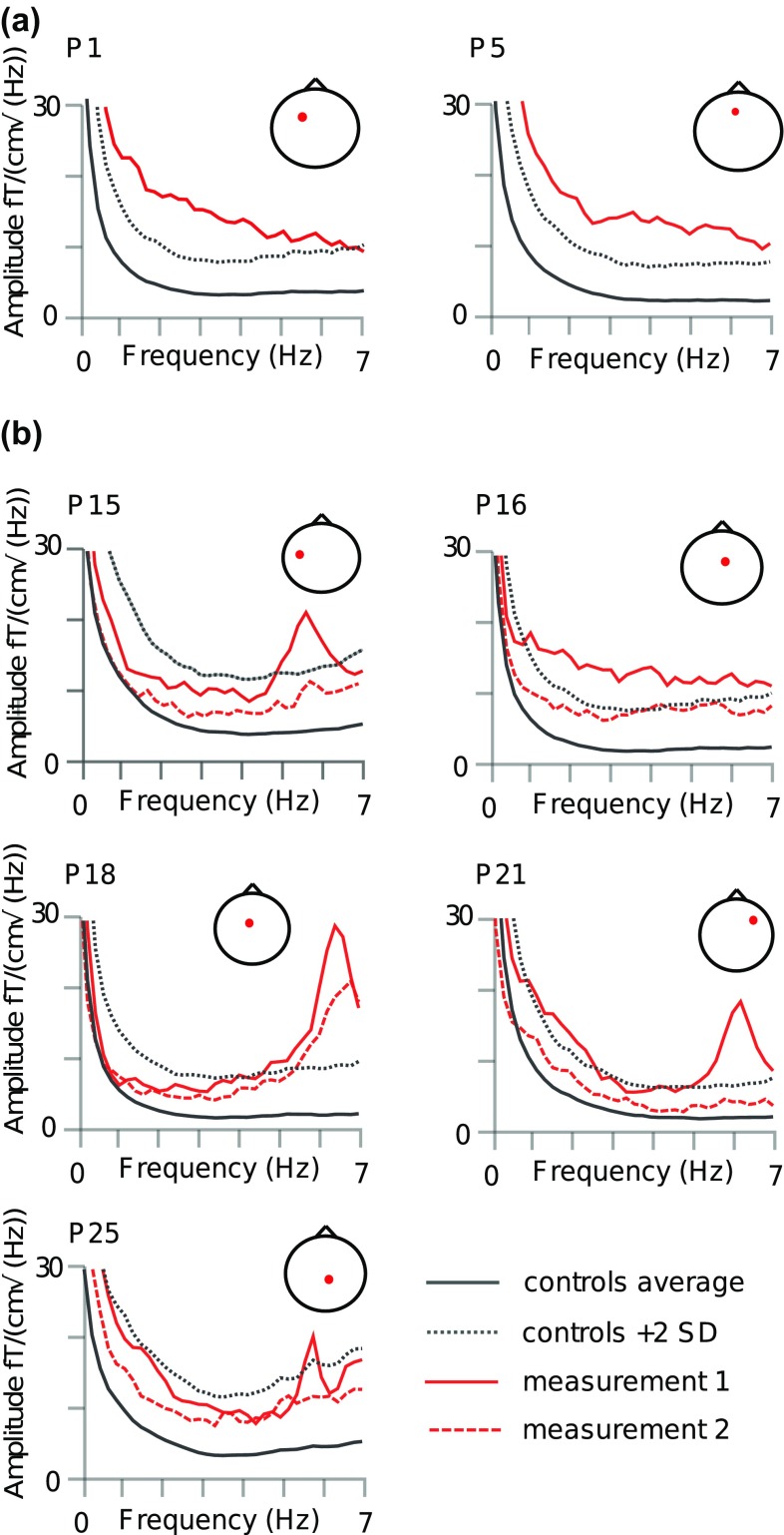
Patients with aberrant low-frequency oscillatory activity. Data at the maximum channel of each patient compared with the average (+ 2 SD) of control subjects. The location of the maximum channel is expressed in the small figure in the upper right corner; nose up, right on right. Note that the average and + 2 SD levels of control data vary between channels. **a** Two patients without repeated measurements **b** five patients with repeated measurements, data from both measurements is shown

Aberrant low-frequency activity exceeding + 2 standard deviation (SD) limit of the controls was visible in 7/26 patients, typically at ~ 4–7 Hz (theta band) (Fig. 1; Table 2), while the same was true only for 2/139 healthy subjects (Chi square statistic 27.6, P = 0.00001). In three of these seven patients, the level of theta activity decreased to normal by the second measurement (patients 15, 21 and 25 in Figs. 1b, 3; Table 2). The difference in the occurrence of low-frequency activity between patients and controls remained significant even at the time of the second measurement (Chi square statistic 16.3; P < 0.0005). The patients were subsequently divided into “acute” (MEG measurements conducted within 1 week post-injury; four patients), “subacute” (MEG at approximately 1 month after injury; nine patients) and “chronic” (MEG later than 1 month post-injury; 24 patients including the second measurements in 12 patients, Table 3). Compared with controls, 1/4 in the acute group (Chi square statistic 10.5; P = 0.004), 3/9 in the sub-acute group (Chi square statistic 26.3; P < 0.0001) and 4/24 patients in the chronic group (Chi square statistic 13.4; P = 0.0008) exhibited aberrant low-frequency activity; The difference was significant at all time-points.

**Table 3:** Subjects with low-frequency activity

Group	Observed	Expected	Chi square statistic	*P* value
Controls (N = 139)	2	2	0	1
Patients				
Acute (N = 4)	1	0.06	10.5	0.004
Subacute (N = 9)	3	0.13	26.3	< 0.0001
Chronic (N = 24)	4	0.35	13.4	0.0008

Observed = the number of subjects presenting low-frequency activity in each subgroup, Expected = the expected number of subjects presenting low-frequency activity, based on the control group data

**Fig. 2:**
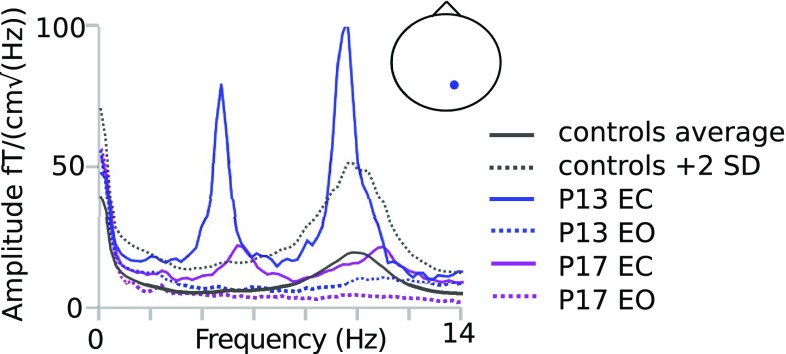
Slow alpha variant activity. Two patients with first low harmonic of alpha activity at ~ 5 Hz compared with the average (+ 2 SD) of healthy controls, depicted in both eyes-closed (EC) and eyes-open (EO) conditions. The small head figure indicates the location of the maximum channel; nose up, right on right

Two additional patients (Fig. 2) showed low-frequency activity at approximately half of their individual alpha frequency over the occipital areas in the eyes-closed condition, and this activity was attenuated when the patients opened their eyes. Similar activity, regarded as ‘slow alpha variant’ (Beauchemin and Savard 2012), was detected in four out of 139 control subjects (Kaltiainen et al. 2016). The occurrence of slow alpha variant activity in the patient group was equivalent to that in the control group (Chi square statistic 1.4, P = 0.23).

**Fig. 3:**
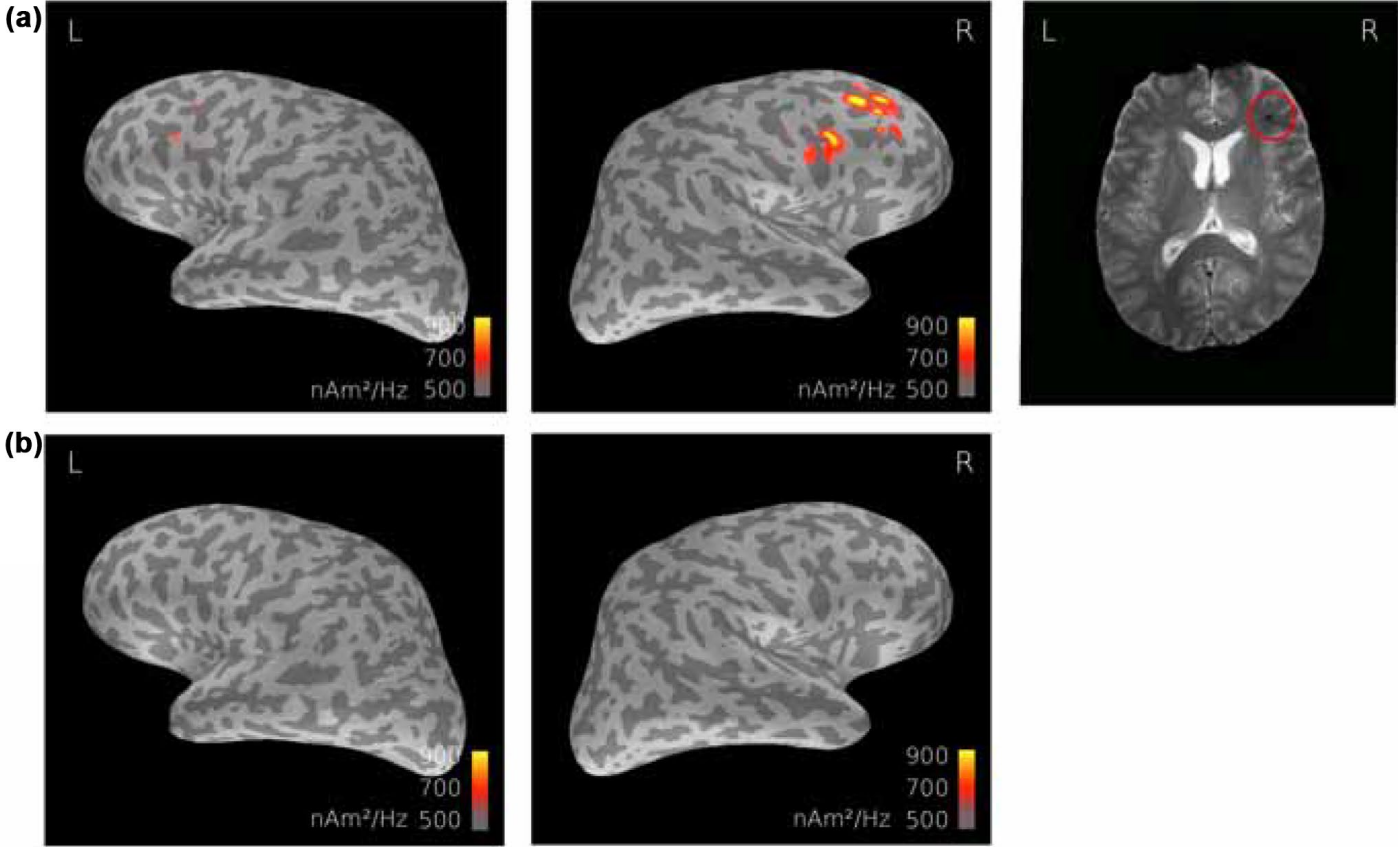
MEG and MRI findings in patient no. 21. **a** MNE dSPMs demonstrate a peak of power spectral density at 6.2 Hz measured 1 week after the trauma (left); T2* MRI of the patient indicated microhaemorrhagia, the red circle indicating the largest lesion in the right frontal lobe (right) **b** No abnormal low-frequency activity could be detected in the same subject 6 months after the trauma, *L* left, *R* right

In each patient with detectable slow frequency activity (0.5–7 Hz), source modelling of the maximum power of the activity agreed with the channel-level measurements and resided in the same area than the MRI lesions (within 3 cm) if observed. Figure 3 depicts the MEG and MRI results in one patient (P21, Table 2; Fig. 1b) with concomitant structural lesions. MEG demonstrated bilateral frontal oscillatory activity with maximum at 6.2 Hz on the right, and 6.1 Hz on the left side, with right hemisphere dominance, modelled to the middle frontal gyrus, measured 1 week after the trauma. Correspondingly, the patient’s structural MRI showed right frontal microhemorrhagia, and a smaller lesion within the left frontal area. In this subject, the abnormal low-frequency activity had disappeared by the time of control measurement at 6 months (Figs. 1, 3b).

Out of the seven patients with abnormal theta activity, three were without any detectable lesions in MRI (Table 2). Over all patients, superficial location of the detected lesion in MRI, i.e. within 3 cm of the cortex convexity, was positively correlated with the occurrence of low-frequency activity in MEG (Chi square statistic 7.2, P < 0.01), whereas deep location (> 3 cm from the cortex convexity) was not (Chi square statistic 2.4, P = 0.12).

### Behavioral Data

In the RPCSQ, the symptom scores varied between 2 and 36 (17 ± 12, average ± SD) at the first measurement, and between 0 and 18 (12 ± 6) at the second measurement. For those measured twice, the symptom score decreased significantly between the two sessions (Z = − 2.2, P < 0.03). The symptom score was not significantly related to the low-frequency activity detected in MEG recordings, nor to MRI lesions (Z = − 0.38, P = 0.7 and Z = − 1.4, P = 0.17, respectively).

## Discussion

Diagnosis of mTBI is currently almost completely clinical, and strongly based on the patient history. Development of objective indicators of trauma with non-invasive methods would be crucial for identifying patients who benefit from closer follow-up and supportive or rehabilitative interventions.

We demonstrate here that relating single-patient resting-state MEG data of mTBI patients to those of healthy adult subjects yields a feasible way for detecting low-frequency cortical activity that can be considered aberrant; such activity was revealed here in 7/26 patients while the same was true in only two out of 139 healthy controls. Increase in low frequency activity has been associated with healthy aging (Gomez et al. 2013). On average, our controls were of younger age than the patients (Table 1). However, the control group included 29 subjects of > 40 years of age, and low-frequency activity exceeding + 2 SD limit was observed only in two female control subjects aged 22 and 25. Thus the observed difference in the occurrence of low-frequency activity between groups is unlikely to stem from the older average age of the patients. Crucially, in three of our patients the low-frequency activity returned to normal limits during the first 6 months, highlighting their partially transient nature and the importance of early timing for the measurements.

Steriade et al. (1990) suggested three probable explanations for aberrant theta activity: slowing of physiological alpha activity, pathological changes in deep midline structures, or mild form of polymorphic delta activity as a sign of axonal damage. The axonal changes may be structural (Oppenheimer 1968; Gloor et al. 1977), or stem from metabolic derangements causing axonal dysfunction and compromising cellular metabolism (Povlishock and Christman 1995). Later on, polymorphic delta activity has been associated with axonal sprouting, and therefore could also represent a repair mechanism after injury (Carmichael and Chesselet 2002). Low-frequency activity has also been located to the areas with peritumoral edema, with mild reduction of *N*-acetyl aspartate and accumulation of lactate as a sign of compromised energy metabolism (Kamada et al. 2001), and found in the perilesional area in patients with cortical strokes possibly related to plastic reorganization of the cortex (Vieth 1990; Butz et al. 2004).

In our study, 3/7 patients with low-frequency activity in MEG were without structural lesions in MRI. MRI was, however, obtained after MEG in one of these cases, which might affect the results (Brandstack et al. 2006). When present, superficial lesions (within 3 cm from the cortical surface of brain convexity) in MRI were significantly correlated with the occurrence of low-frequency MEG activity, probably reflecting the above-mentioned perilesional low-frequency activity (Table 2). In comparison, only one out of seven patients with detected pathological MEG activity had deep MRI lesions affecting white-matter tracts. In this case, the observed slow-wave activity had a mixed frequency distribution (Table 2), possibly as a result of cortical deafferentation as discussed above. The detectability of current sources decreases with source depth, and more so for MEG than EEG recordings (Goldenholz et al. 2009; Hunold et al. 2016), and combination of EEG and MEG measurements in these patients would likely provide better identification of pathological activity.

Here, two patients, and in our earlier study four out of 139 healthy controls, exhibited low-frequency activity that reacted to eye-opening, and thus likely represented a non-pathological alpha variant. Such an interpretation can only be drawn from successive eyes-closed versus eyes-open reactivity measurements, which are thus highly recommended when assessing low-frequency activity in these patients.

Swan et al. demonstrated significant correlations between MEG low-frequency activity and cognitive tests assessing executive functioning and processing speed at 3 months after mTBI (Swan et al. 2015). The performance of these patients, however, was within clinically normal limits. In our patients, the occurrence of low-frequency activity was not significantly related to the severity of symptoms as measured by RPCSQ score. It is well known that post-concussion symptoms are not specific to traumatic brain injury, and similar symptoms are frequently detected in patients with e.g. chronic pain, depression and post-traumatic stress disorder (Smith-Seemiller et al. 2003; Stalnacke 2012, Bryant 2011). A high RPCSQ score evaluated at the emergency department correlates with the hospital admission, re-admission within 30 days after discharge, and with post-concussion syndrome at early follow-up but not later (Ganti et al. 2016). It is probable that RPCSQ was not a sensitive enough measure for our patients, some of who had only mild symptoms already at the time of the first MEG measurement. However, our results show that even in patients with very mild behavioral problems after the trauma, functional imaging can demonstrate significant early changes, thus warranting closer follow-up.

Earlier MEG-studies assessing low-frequency activity in mTBI have typically been conducted months or even years after the trauma in perpetually symptomatic patients (Huang et al. 2009; Lewine et al. 1999, 2007). This may be the main contributing factor to the different detection rates of low-frequency activity: some of our patients were already well recuperated at the time of recordings, one having RPCSQ score two with basically no behavioral symptoms at all. Our detection method, i.e. comparison of each individuals’ oscillatory power at different frequency bands to that of large control group of healthy subjects, may be sensitive to false negatives due to individual differences in oscillatory power. However, the method is clinically feasible and easily managed, and the method was shown to be efficient in the present patient group with mild behavioral symptoms.

Our results on the transient nature of the detectable low-frequency activity in a considerable number of patients is thus of great interest and opens up the possibility for assessing the potential long-term symptoms of these patients prospectively. Furthermore, it encourages selection of patients with detectable pathological activity for a closer follow-up and rehabilitative interventions when needed. Indeed, transcranial direct current stimulation (tDCS) has resulted in significant reduction of delta-activity in TBI patients, and the efficacy of the treatment correlates with improved performance in neuropsychological testing (Ulam et al. 2015); EEG has been suggested as the natural potential biomarker for assessing such changes (Ulam et al. 2015; Li et al. 2015). Interestingly in a recent study, patients with excess low-frequency activity in initial recordings exhibited greater improvement in neuropsychological tests after active tDCS intervention, compared with patients without low-frequency activity receiving active tDCS intervention, and sham tDCS groups (Ulam et al. 2015); this finding might also reflect the possible neuronal repair nature of low-frequency activity.

Differences in local and overall connectivity measures within the resting state network have also been reported between mTBI and control subjects. Zouridakis et al. (2012) found a deficient local MEG connectivity network encompassing centroparietal regions, and a reduction in the overall connectivity in mTBI patients 3 months after the trauma. At a similar timeline, Dunkley et al. (2015) reported significant alterations in correlations within several frequency bands in mTBI patients with respect to controls. Dimitriadis et al. (2015) demonstrated diminished local connectivity combined with enhanced long-distance connectivity in mTBI patients compared with controls. These approaches, however, require laborious analyses and special technical proficiency, thus hampering their clinical use in the majority of sites.

Despite strenuous research in this field, prospective indicators that could be used for prediction of recovery are scarce. The challenge lies in finding measures that are robust at individual level to aid in clinical decision making. The population of this study was heterogeneous, which is also true for real clinical patients. Detection of theta activity could serve in selecting patients with objective functional changes in MEG/EEG, for further observation or rehabilitation after mild traumatic brain injury. Comparison of MEG/EEG oscillatory activity between patients and controls provides a clinically feasible method for detecting pathological low-frequency, which seems to be easiest to detect for superficial lesions, and when measured shortly after trauma. We suggest that sensor-level MEG or EEG measures taken early within the first weeks after mTBI could be considered as a relatively easy way to screen patients with major symptoms but negative structural MRI, as they may support clinical decision making in these patients.

Future studies would benefit from larger sample size, prospective design, stable timing of repeated MEG recordings and structural imaging after trauma, to further assess the natural evolution of theta-band activity, its’ prognostic value, and its relationship to structural lesions. In addition to objective standardized neuropsychological testing, assessment of depression and anxiety, as well as coping strategies in the subacute phase after mTBI, might help in targeting patients at risk for incomplete recovery (van der Naalt et al. 2017). In the future, observed theta-activity could also serve in selecting patients for rehabilitation interventions, such as tDCS, if a firm relationship of initial theta-band activity and favorable response to tDCS is confirmed.

## Conclusions

We found aberrant low-frequency MEG activity in 7/26 patients compared with 2/139 healthy subjects early after mTBI. In some patients, the pathological activity was of transient nature, being visible only in our first recordings within 6–26 days after the injury. Our results suggest that MEG, or EEG, could be used for objective detection of brain dysfunction in mTBI patients with extensive symptoms and negative MRI, and emphasize prompt timing for the measurements in clinical settings. The possible relationship of very early oscillatory changes with post-concussion symptoms and rehabilitation interventions needs to be further investigated with larger patient groups and detailed neuropsychological evaluation closely timed with the measurements.
